# High precision visual localization method of UAV based on feature matching

**DOI:** 10.3389/fncom.2022.1037623

**Published:** 2022-11-09

**Authors:** Bayang Xue, Zhong Yang, Luwei Liao, Chi Zhang, Hao Xu, Qiuyan Zhang

**Affiliations:** ^1^Nanjing Taiside Intelligent Technology Co., Ltd., Nanjing, China; ^2^College of Automation Engineering, Nanjing University of Aeronautics and Astronautics, Nanjing, China; ^3^School of Mathematics and Physics, Anhui University of Technology, Ma'anshan, China; ^4^Guizhou Electric Power Research Institute, Guizhou Power Grid Co., Ltd., Guiyang, China

**Keywords:** UAV, AKAZE, image matching, localization and orientation, hovering

## Abstract

In this paper, the precision hovering problem of UAV operation is studied. Aiming at the diversity and complexity of the UAV operating environment, a high-precision visual positioning and orientation method based on image feature matching was proposed. The image feature matching based on the improved AKAZE algorithm is realized, and the optimal matching point pair screening method based on the fusion of Hamming distance and matching line angle is innovatively proposed, which greatly improves the robustness of the algorithm without affecting the performance of the algorithm. The real-time image is matched with the benchmark image for image feature matching. By reducing the deviation of image feature, the pose state correction of UAV hovering is achieved, and the precision hovering of the UAV is realized. Both simulation and real UAV tests verify the effectiveness of the proposed UAV high-precision visual positioning and orientation method.

## Introduction

In the inspection process of bridges, towers, transmission lines, and other facilities, unmanned aerial vehicles (UAVs) often need to hover at fixed points for key parts. The stability of the UAVs hovering will greatly affect the quality of the operation, and even affect the smooth completion of the operation. At present, UAVs mainly rely on the inertial navigation system and the global navigation satellite system's (GNSS) navigation and positioning system. Due to the accumulation of errors in the inertial navigation system, the GNSS is affected by signal strength and accuracy is limited, and the UAVs are hindered by external wind gusts, which often leads to pose deviation and is difficult to correct.

Using traditional navigation methods, it is difficult to achieve high-precision fixed-point hovering of UAVs. At present, many UAVs' pose guidance through machine vision has been carried out at home and abroad. The fixed-point hovering control of small quadrotor UAVs based on optical flow and ultrasonic module is mentioned in literature (Zhang et al., 2018). However, the optical flow algorithm will be disturbed by the slight shaking of ground objects and the change of light and shade, and the robustness of the algorithm is poor. Literature (Yu et al., 2021) proposed the indoor fixed-point hovering method of UAVs based on the pyramid optical flow method. This method can only obtain the horizontal moving speed of the UAV, and its height needs to depend on the barometer, so the algorithm has poor robustness in corridors and other scenes. Wang et al. (2019) proposed a fixed-point hovering method for a quadrotor based on the Harris algorithm. The corner detection method was used to obtain the overlapping area of the image to calculate the UAV height. However, the corner detection method is not scale invariant and is only suitable for hovering control of the UAV under small disturbances. Therefore, this paper proposes an image matching method based on image scale-invariant features for UAV pose guidance during hovering operation.

**Figure 1:**
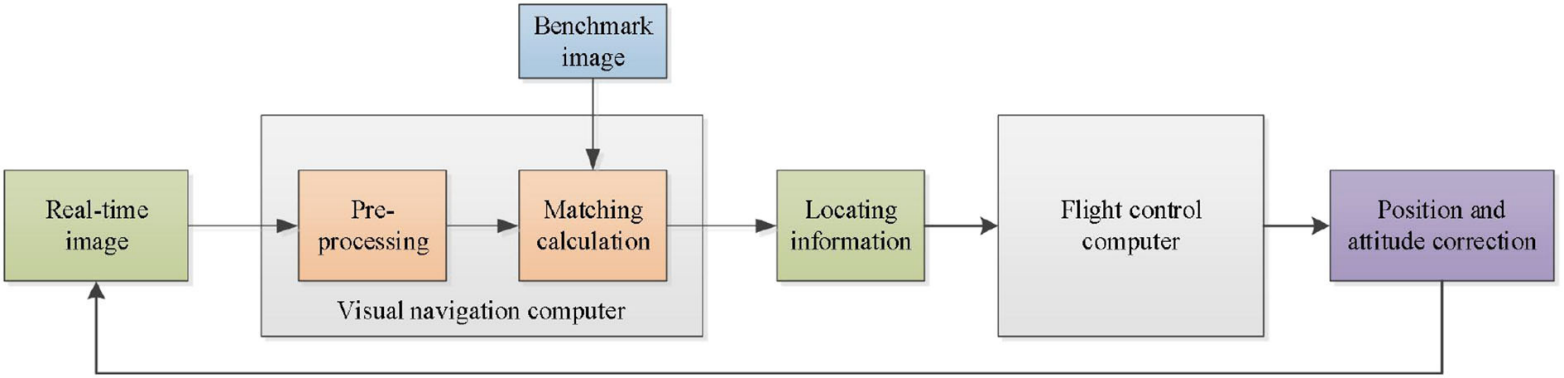
UAV Visual guidance system based on feature matching.

As shown in Figure 1, the schematic diagram of the UAV visual guidance system is based on feature matching. In UAV operation, the reference image is set as the reference matching object, and the visual navigation computer preprocesses the real-time image, further extracts image features, and matches image features with the reference image. The positioning information of the real-time image relative to the reference image, including translation, rotation, and scaling, is calculated and entered into the flight control computer as the pose compensation parameters to correct the UAV pose. By reducing the feature deviation between the real-time image and the reference image, accurate visual guidance can be achieved when the UAV is hovering.

During the flight of the UAV, the dynamic images are often affected by rotation, scale scaling, brightness change, angle of view change, affine transformation, noise, and so on. In this case, to achieve better image matching, it is necessary to find an image matching algorithm that can overcome these disturbances. In order to satisfy the scale invariance of the feature matching algorithm, it is necessary to construct different scale spaces for feature extraction. The main construction methods of scale space are as follows: (1) convolution of gaussian kernel function with gray image; and (2) construct by using a nonlinear filtering function.

The matching algorithms that use the Gaussian kernel function to construct scale space include the SIFT algorithm (Bellavia, 2022; Liu et al., 2022), SURF algorithm (Liu et al., 2019a; Liu Z. et al., 2021; Fatma et al., 2022), and ORB algorithm (Liu et al., 2019b; Chen et al., 2022; Xie et al., 2022; Xue et al., 2022), etc. This kind of algorithm has good robustness and fast matching speed, but the Gaussian kernel convolution operation will lead to the loss of edge information of the image, which seriously affects the stability of feature points and descriptors. The main algorithms that use nonlinear filtering function to construct scale space include the KAZE algorithm (Khalid et al., 2021; Liu J.-B. et al., 2021; Roy et al., 2022), AKAZE algorithm (Sharma and Jain, 2020; Ji et al., 2021; Pei et al., 2021; Yan et al., 2021), etc. The scale space constructed by the nonlinear filtering function can better protect the information at the edge of the image and increase the robustness of the matching algorithm. The feature point detection and descriptor of the KAZE algorithm are all borrowed from the SURF algorithm, but the robustness of the KAZE algorithm is stronger than that of the SURF algorithm, which is enough to prove the superiority of nonlinear scale space. AKAZE (Accelerated-KAZE) is an improved feature point detection and description algorithm proposed by KAZE at the 2012 ECCV Conference. AKAZE improves the KAZE algorithm in two main ways:

(1) The fast explicit diffusion (FED) framework is introduced to solve partial differential equations. The scale space established by FED is faster than other nonlinear models, and more accurate than the airborne optical sectioning (AOS) method;
(2) An efficient modified local difference binary descriptor (M-LDB) is introduced to improve the rotation and scale invariant robustness compared with the original LDB, and the scale spatial gradient information constructed by FED is combined to increase the uniqueness.

Compared with the SIFT and SURF algorithms, the AKAZE algorithm is faster. Meanwhile, compared with the BRISK (Niyishaka and Bhagvati, 2020; Singh and Singh, 2020; Shi et al., 2021), FAST (Feng et al., 2021; Yang et al., 2022; Zhang and Lang, 2022) and ORB algorithms, the repeatability and robustness are greatly improved. The descriptors obtained by the AKAZE's feature algorithm have rotation invariance, scale invariance, illumination invariance, space invariance, etc., and have high robustness, feature uniqueness, and feature accuracy. In view of the real-time requirements of the algorithm and the characteristics of rotation, scaling, and translation of the UAV operation images, this paper studies the UAV visual navigation (Cao et al., 2022; Guo et al., 2022; Qin et al., 2022; Zhao et al., 2022) algorithm based on the improved AKAZE algorithm, especially the precise hovering problem in UAV operation.

## Visual navigation algorithm design

### Visual navigation based on improved AKAZE algorithm

#### Construct nonlinear scale space

First, it is necessary to carry out nonlinear diffusion filtering on the image. The advantage of a nonlinear diffusion filtering (Li et al., 2016; Feng and Chen, 2017; Jubairahmed et al., 2019; Liu et al., 2019c) algorithm is that it can filter the image noise while preserving important boundary and other details. The nonlinear diffusion filtering algorithm is mainly a diffusion process in which the gray image changes at different scales are expressed as flow functions. The nonlinear partial differential equation can be expressed by Equation (1).


(1) $$\begin{array}{l} \frac{\partial L}{\partial \text{t}}=div\left(c\left(x,y,t\right)\nabla L\right) \end{array}$$

where, *L* represents gray image information, *div* represents diffusion of flow function, ∇ represents image gradient, and *c*(*x, y, t*) is conduction function. The time parameter *t* corresponds to the scale factor, which is controlled by the image gradient size during the diffusion process. The conduction function formula is defined by Equation (2).


(2) $$\begin{array}{l} c\left(x,y,t\right)=g\left(\left|\nabla L_{\sigma }\left(x,y,t\right)\right|\right) \end{array}$$

where, ∇*L*_σ_ represents the gradient image of gray image *L* after Gaussian filtering. The conduction kernel function is selected optimally for regional diffusion smoothing, as shown in Equation (3).


(3) $$\begin{array}{l} g=\frac{1}{1+\frac{{\left|\nabla L_{\sigma }\right|}^{2}}{\lambda^{2}}} \end{array}$$

where, the parameter λ is the contrast factor controlling the diffusivity, and the decision factor determining the enhancement and flat region filtering in the edge region.

The AKAZE algorithm constructs nonlinear scale space in a similar way to the SIFT algorithm, both of which need to set groups *o* and layers *s*. The calculation formula of image scale parameters σ_*i*_ is expressed by Equation (4).


(4) $$\begin{array}{l} \sigma_{i}\left(o,s\right)=2^{\frac{o+s}{s}} \end{array}$$

The scope of the variable can be expressed by Equation (5).


(5) $$\begin{array}{l} o\in \left[0,1,\ldots ,O-1\right],s\in \left[0,\ldots ,S-1\right],i\in \left[0,\ldots ,M-1\right] \end{array}$$

where, *o* is the number of groups, is the number of layers in each group, and *M* is the total number of filtered images. The scale parameters σ of nonlinear scale space are converted into time units, and the mapping relationship can be expressed by Equation (6).


(6) $$\begin{array}{l} t_{i}=\frac{1}{2}\sigma_{i}^{2},i=0\ldots M \end{array}$$

The core idea of the FED algorithm is to change the step size τ_*j*_ of *n* explicit diffusion processes to carry out *M* steps of the cycle. The formula for calculating the step size τ_*j*_ is expressed by Equation (7).


(7) $$\begin{array}{l} \tau_{j}=\frac{\tau_{\max}}{2\cos^{2}\frac{\pi \left(2j+1\right)}{\left(4n+2\right)}} \end{array}$$

In the formula, τ_max_ is the maximum step size threshold, and in order to ensure the stability of the constructed scale space, τ is smaller than τ_max_, and τ_max_ represents the maximum iteration step that does not destroy the stability of the explicit equation. Equation (1) above can be expressed by a vectorization matrix, as shown in Equation (8).


(8) $$\begin{array}{l} \frac{L^{\left(i+1\right)}-L^{i}}{\tau }=A\left(L^{i}\right)L^{i} \end{array}$$

where, *A*(*L*^*i*^) is the conduction matrix for image coding, and τ is a constant time step. In the explicit method, the solution of *L*^(*i*+1)^ will be directly calculated through the previous image evolution *L*^*i*^ and image conduction function *A*(*L*^*i*^), as shown in Equation (9).


(9) $$\begin{array}{l} L^{\left(i+1,j+1\right)}=\left(I+\tau_{j}A\left(L^{i}\right)\right)L^{\left(i+1,j\right)},j=0,1,\ldots ,n-1 \end{array}$$

The matrix *A*(*L*^*i*^) remains the same throughout the FED loop. When the FED loop ends, the algorithm recalculates the matrix *A*(*L*^*i*^).

#### Feature point detection and localization

**Figure 2:**
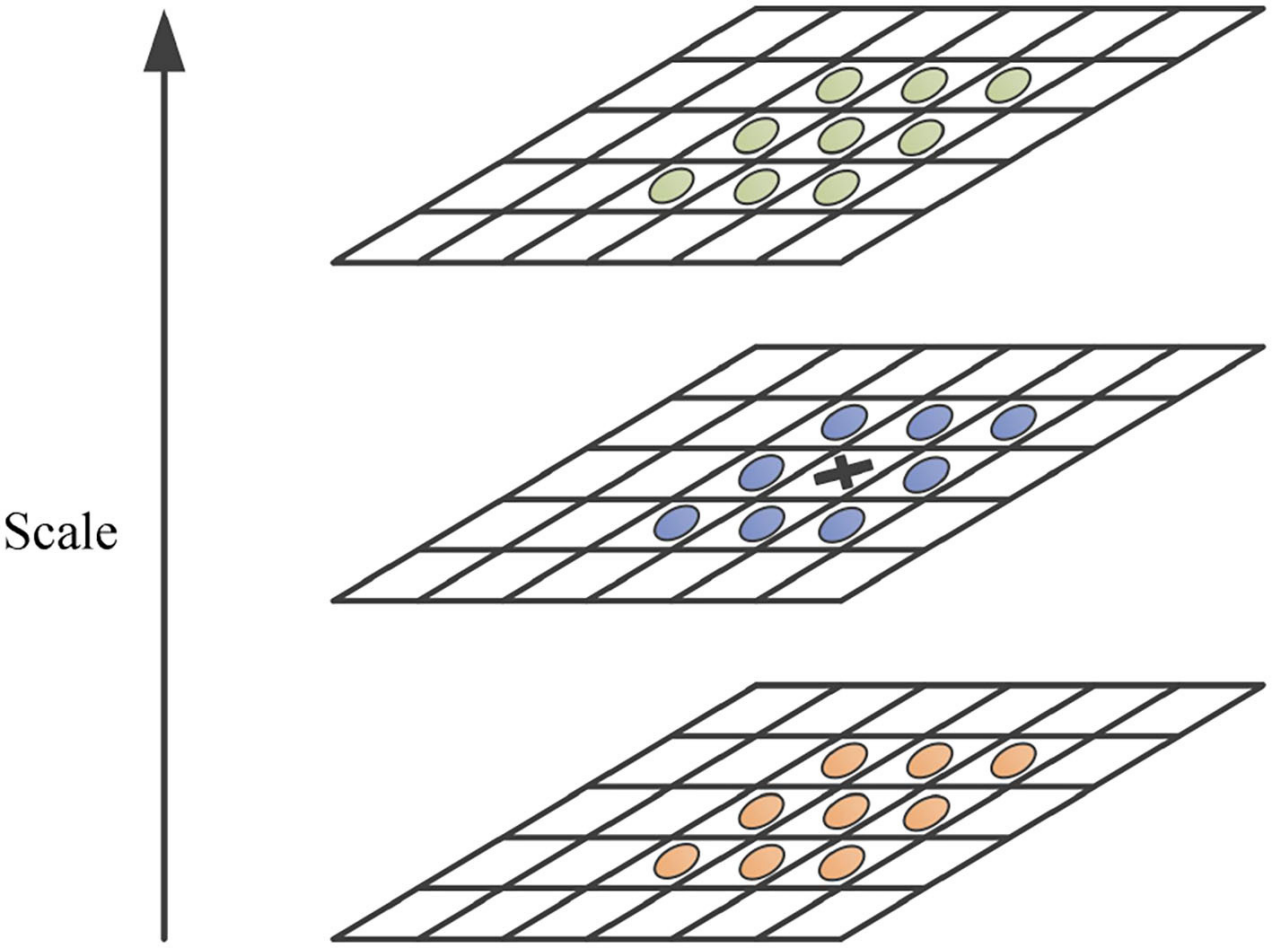
Location feature point.

After building the scale space, it is necessary to further detect the feature points. By calculating the determinant of the Hessian for each filtered image *L*^*i*^ in the non-linear scale space, the normalized differential multiscale operator $\sigma_{i}^{2}$ is used to find the maximum point of the determinant of the Hessian matrix. As shown in Figure 2, the response value of the sampling point is compared with eight neighborhood points in the same scale and 2 × 9 neighborhood points in adjacent scales to determine whether it is the maximum value.

The determinant of the Hessian matrix is expressed by Equation (10).


(10) $$\begin{array}{l} L_{Hessian}^{i}=\sigma_{i}^{2}\left(L_{xx}^{i}L_{yy}^{i}-L_{xy}^{i}L_{xy}^{i}\right) \end{array}$$

where, σ_*i*_ is the initial value of scale factor; $L_{xx}^{i}$ is the second transverse derivative; $L_{yy}^{i}$ is the second longitudinal derivative; $L_{xy}^{i}$ is the second cross differential.

#### Find the principal direction of the feature points

**Figure 3:**
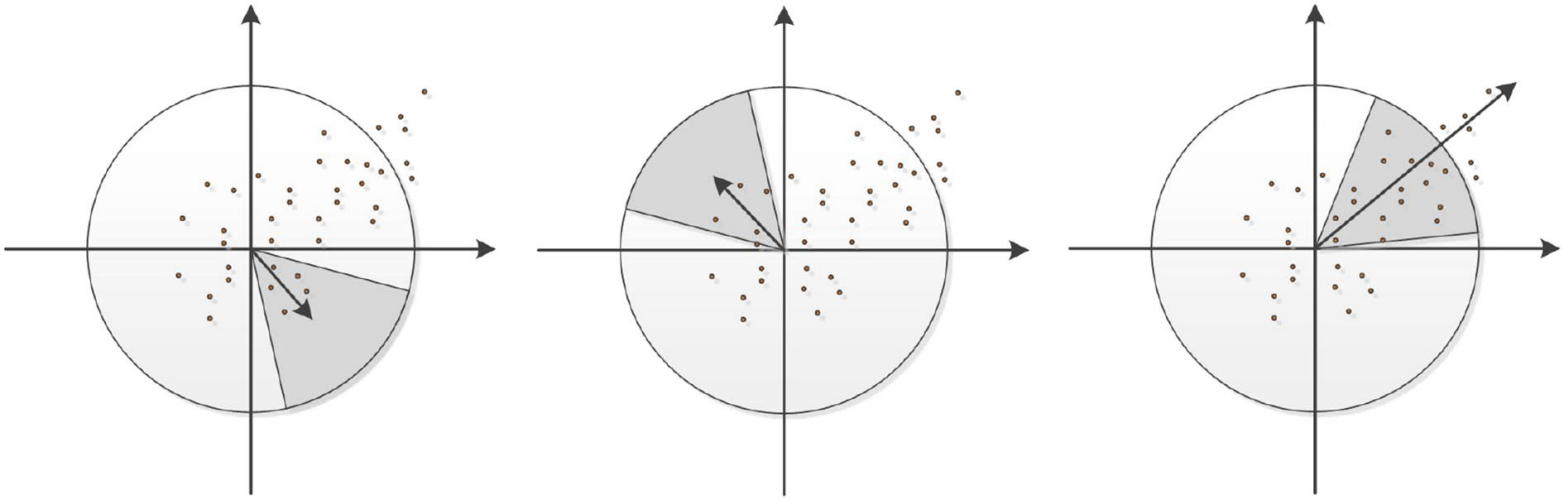
Selection of principal direction of feature points.

After locating the feature points, the principal direction of the feature points should be further solved. As shown in Figure 3, the circular area is determined with the feature points as the center and 6σ as the radius, and the first-order differential values *L*_*x*_ and *L*_*y*_ of the points in the neighborhood of the feature points are calculated respectively, and the Gaussian weighting operation is carried out. Rotate the fan window with angle π/3, superimpose the vectors of points in the neighborhood, and select the direction of the longest vector in the sum of unit vectors as the main direction. Then rotate the sector window around the origin, recalculate all the Gaussian weighted vectors in the sector area after rotation, and repeat this operation until the whole circular area is counted. Finally, the direction represented by the sector region with the highest superposition value is taken as the principal direction of the feature point.

#### Construct the M-LDB descriptor

After extracting the feature points in the nonlinear scale space and establishing the principal direction of the feature points, the feature points are described by the M-LDB descriptor. The specific construction method is as follows:

In the first step, the M-LDB descriptor is constructed by selecting an appropriate sampling region *P* centered on the feature points, and the sampling region *P* is divided into *n* × *n* meshes. The average value of all pixels in a *n* × *n* single grid is calculated by Equation (11).


(11) $$\begin{array}{l} I_{avg}\left(i\right)=\frac{1}{m_{i}}\overset{m_{i}}{\underset{k=1}{\sum }}I\left(k\right) \end{array}$$

where, *I*(*k*) is the pixel value of the gray image; *m* is the number of pixels in each partition grid; *i* is the number of grids that the sampling area is divided into.

In the second step, the gradients of pixels in each grid in the *x* and *y* directions are calculated and encoded according to the average pixel value of each grid and the gradient value of grid pixels, which can be expressed by Equation (12).


(12) $$\begin{array}{l} Func\left(•\right)=\left\{Func_{\text{int }ensity}\left(i\right),Func_{dx}\left(i\right),Func_{dy}\left(i\right)\right\} \end{array}$$

where, *Func*_int *ensity*_ (*i*) = *I*_*avg*_ (*i*),*Func*_*dx*_ (*i*) = *Gradient*_*x*_ (*i*) = *dx*,*Func*_*dy*_ (*i*) = *Gradient*_*y*_ (*i*) = *dy*.

In the third step, it can be obtained from the above equation that the LDB descriptor compares the average intensity and gradient between paired grid cells respectively and is set to 0 or 1 according to the comparison results. The formula is expressed by Equation (13).


(13) $$\tau \left(Func\left(i\right),Func\left(j\right)\right)=\{\begin{array}{c} 1\;\left(Func\left(i\right)-Func\left(j\right)\right)>0\\ 0\text{ other} \end{array}$$

Then the M-LDB descriptor can be expressed by Equation (14).


(14) $$\begin{array}{l} f_{n}=\underset{1\leq i<N}{\sum }2^{i-1}\tau \left(Func\left(i\right),Func\left(j\right)\right) \end{array}$$

#### Descriptor matching and image localization

Since the M-LDB is a binary descriptor, Hamming distance is used to calculate the similarity of descriptors. Hamming distance can calculate the distance of binary descriptors only through XOR operation, so the matching efficiency of feature points is high. In addition, in order to improve the matching accuracy of feature points, the homography matrix between two images is calculated by the RANSAC algorithm, and the wrong matching is eliminated to obtain accurate matching results of feature points. The Hamming distance can be calculated by the following Equation (15).


(15) $$\begin{array}{l} dist\left(\vec{a},\vec{b}\right)=\underset{i}{\sum }\left(a_{i}==b_{i}\right)?1:0 \end{array}$$

After the aforementioned feature point matching and screening, the correctly matched feature point pairs can be obtained, and the motion parameters of the real-time image and the benchmark image can be correctly estimated through these feature point pairs. In this paper, affine transformation is used to model the motion between images, which is defined by Equation (16).


(16) $$\begin{array}{l} \left(\begin{array}{c} x\\ y\\ 1 \end{array}\right)=H\left(\begin{array}{c} x\prime \\ y\prime \\ 1 \end{array}\right) \end{array}$$

where, $H=\left(\begin{array}{ccc} a_{11} & a_{12} & a_{13}\\ a_{21} & a_{22} & a_{23}\\ 0 & 0 & 1 \end{array}\right)$ is the affine transformation matrix, also known as the homography matrix, with 6 degrees of freedom; *a*_13_ and *a*_23_ are used to describe the translation change between images; *a*_11_, *a*_12_, *a*_21_, and *a*_22_ are used to describe rotation and scaling changes between images; (*x′, y′*) represents the pixels of the previous image; (*x, y*) represents the pixels of adjacent images after affine transformation.

#### Matching point selection based on hamming distance and matching line angle fusion

In this paper, the Hamming distance is considered as a preliminary method to eliminate mismatches, and an adaptive threshold method is proposed for different scenes. The specific steps are as follows:

(1) First, the matching points are sorted according to their Hamming distance to obtain the minimum Hamming distance.
(2) The threshold is set as *d* = *n* × *d*_min_, and all feature point matching points are traversed. The Hamming distance of each feature point is compared with the threshold *d*. The feature matching point pairs with a Hamming distance less than *d* are returned and saved as sub-optimal matching points. Here, the value of *n* needs to be adjusted according to different scenes. In this paper, by establishing the threshold library of scenes, the algorithm automatically loads the adaptive threshold parameters through scene analysis to realize the effective compatibility of the algorithm in multiple scenes.

After the screening of the Hamming distance, the screening effect is often not good, and there will be some obviously wrong matching points. Through a large number of experiments, it is found that the wrong matching lines often have a large deviation angle from most of the correct matching lines. This paper innovatively proposes that the matching line with the shortest Hamming distance is taken as the benchmark to calculate the angle between other matching lines and the reference line and filters the wrong matching point pairs by setting an*g*_max_. The correct matching condition is set by Equation (17).


(17) $$\begin{array}{l} \left(ang<ang_{\max}\;\&\;\&\;dit<d\right) \end{array}$$

Through repeated experimental verification, the robustness of the algorithm is greatly improved without affecting the performance of the algorithm, which lays a theoretical foundation for the engineering application of the algorithm. The angle between the matching line *l*_2_ and the reference line *l*_1_ is deduced as follows:

The equation of the line *l*_1_ is expressed by Equation (18).


(18) $$\begin{array}{l} a_{1}x+b_{1}y+c_{1}=0 \end{array}$$

The equation of the line *l*_2_ is expressed by Equation (19).


(19) $$\begin{array}{l} a_{2}x+b_{2}y+c_{2}=0 \end{array}$$

Then the intersection point of *l*_1_ and *l*_2_ is calculated by Equation (20).


(20) $$\{\begin{array}{l} a_{1}x+b_{1}y+c_{1}=0\\ a_{2}x+b_{2}y+c_{2}=0 \end{array}\Rightarrow \{\begin{array}{l} x=\frac{b_{1}c_{2}-b_{2}c_{1}}{a_{1}b_{2}-a_{2}b_{1}}\\ y=\frac{a_{1}c_{2}-a_{2}c_{1}}{a_{2}b_{1}-a_{1}b_{2}} \end{array}$$

The intersection point *p*_0_ (*x*_0_, *y*_0_), the end point *p*_2_ (*x*_2_, *y*_2_) of *l*_1_, and the end point *p*_4_ (*x*_4_, *y*_4_) of *l*_2_ form a triangle, then the length of each side is expressed by Equation (21), Equation (22) and Equation (23).


(21) $$\begin{array}{l} a=\sqrt{{\left(x_{4}-x_{2}\right)}^{2}+{\left(y_{4}-y_{2}\right)}^{2}} \end{array}$$

(22) $$\begin{array}{l} b=\sqrt{{\left(x_{4}-x_{0}\right)}^{2}+{\left(y_{4}-y_{0}\right)}^{2}} \end{array}$$

(23) $$\begin{array}{l} c=\sqrt{{\left(x_{2}-x_{0}\right)}^{2}+{\left(y_{2}-y_{0}\right)}^{2}} \end{array}$$

The angle θ is derived from the law of cosines, as shown in Equation (24).


$$\begin{array}{l} a^{2}=b^{2}+c^{2}-2bc\cos\theta \Rightarrow \cos\theta =\frac{b^{2}+c^{2}-a^{2}}{2bc}\Rightarrow \theta \end{array}$$

(24) $$\begin{array}{l} =\arccos\left(\frac{b^{2}+c^{2}-a^{2}}{2bc}\right) \end{array}$$

#### UAV position and attitude modification

**Figure 4:**
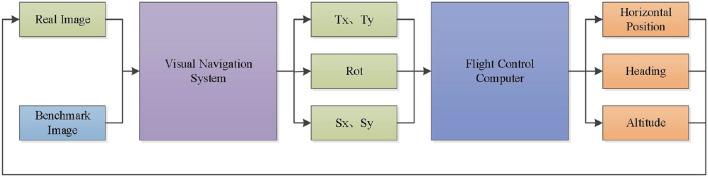
Schematic of UAV visual navigation system.

The image matching and positioning are realized, and the deviation of image features needs to be further calculated to form data connection with the flight control computer. Figure 4 shows the schematic diagram of the high-precision visual positioning system in this paper. The real-time image collected by the UAV and the set benchmark image are used as the input end of the visual navigation module. After the image matching and positioning of the visual navigation system, the translation, rotation, and scaling of the real-time image relative to the benchmark image are output. Taking the amount of translation, rotation, and scaling as the input parameters of the flight controller, the deviation between the real-time image and the reference image can be reduced by modifying the horizontal position, heading, and altitude of the UAV, so as to realize the closed-loop control of the UAV visual positioning and orientation.

**Figure 5:**
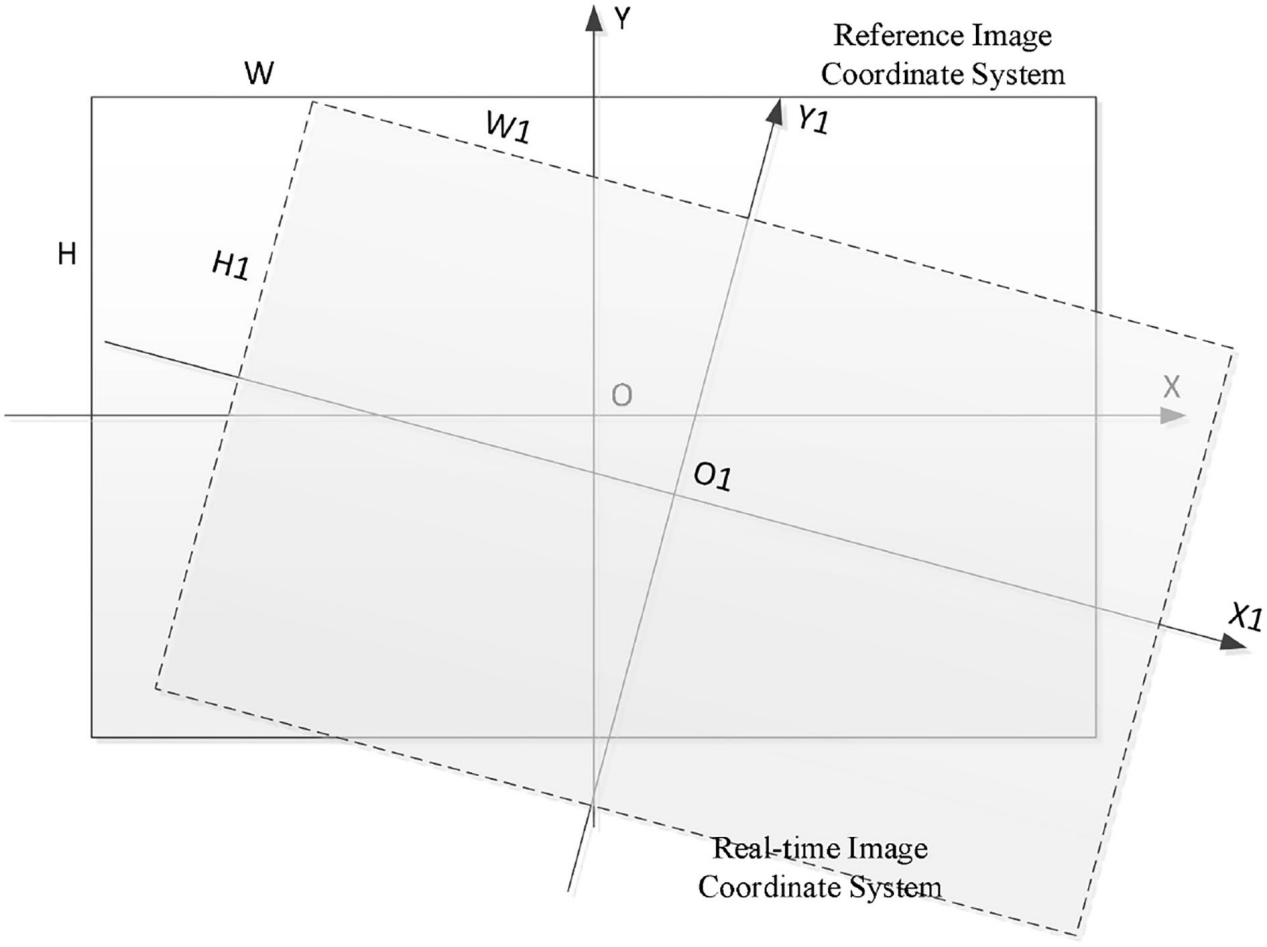
Deviation of the real-time image from the reference image.


As shown in Figure 5, is the deviation diagram between the real-time image and the reference image. Tx is the X-axis offset of UAV in the reference image coordinate system; is the Y-axis offset of UAV in the reference image coordinate system; *Tx* and *Ty* are input quantities for UAV plane position correction. *Rot* is the rotation angle of the real-time image relative to the reference image, and *Rot* is the input of the UAV heading correction. *Sx* and *Sy* are the scaling ratio of the real-time image relative to the benchmark image, and *Sx* and *Sy* are the input quantities for UAV height correction. By reducing the offset of image features, the UAV 3D pose correction can be realized, and high-precision visual positioning and orientation can be realized. *Tx* and *Ty*can be calculated by Equation (25).

(25) $$\begin{array}{l} \left(Tx,Ty\right)=O_{1}\left(x,y\right)-O\left(x,y\right) \end{array}$$

From the formula of the angle between vectors, it can be known that, as shown in Equation (26).


(26) $$\begin{array}{l} \cos Rot=\frac{\vec{OX}\times \vec{O_{1}X_{1}}}{\left|\vec{OX}\times \vec{O_{1}X_{1}}\right|} \end{array}$$

Then, the rotation angle of the real-time image relative to the reference image can be calculated by Equation (27).


(27) $$\begin{array}{l} Rot=\arccos\frac{\vec{OX}\times \vec{O_{1}X_{1}}}{\left|\vec{OX}\times \vec{O_{1}X_{1}}\right|} \end{array}$$

Sx is the scaling quantity of the real-time image relative to the reference image in the width direction, and *Sy* is the scaling quantity of the real-time image relative to the reference image in the length direction. The calculation formula is expressed by Equation (28).


(28) $$\begin{array}{l} Sx=W_{1}/W,Sy=H_{1}/H \end{array}$$

## System algorithm flow

This system uses the improved AKAZE image matching algorithm for the precise hovering of UAV operation. The algorithm flow of the system is as follows:

(1) Set the reference image of UAV operation;
(2) The key points of the benchmark image and real-time image are detected respectively;
(3) M-LDB descriptors of key points of two images are calculated;
(4) BruteForce-Hamming is used to match descriptor vectors;
(5) The best matching points are selected based on the fusion of Hamming distance and matching line angle;
(6) The RANSAC algorithm is used to calculate the homography matrix between two images;
(7) The feature deviation and position information of the real-time image relative to the reference image are calculated;
(8) The rotation, translation, and scaling of the real-time image relative to the reference image are calculated and input to the flight controller for UAV pose correction.

**Figure 6:**
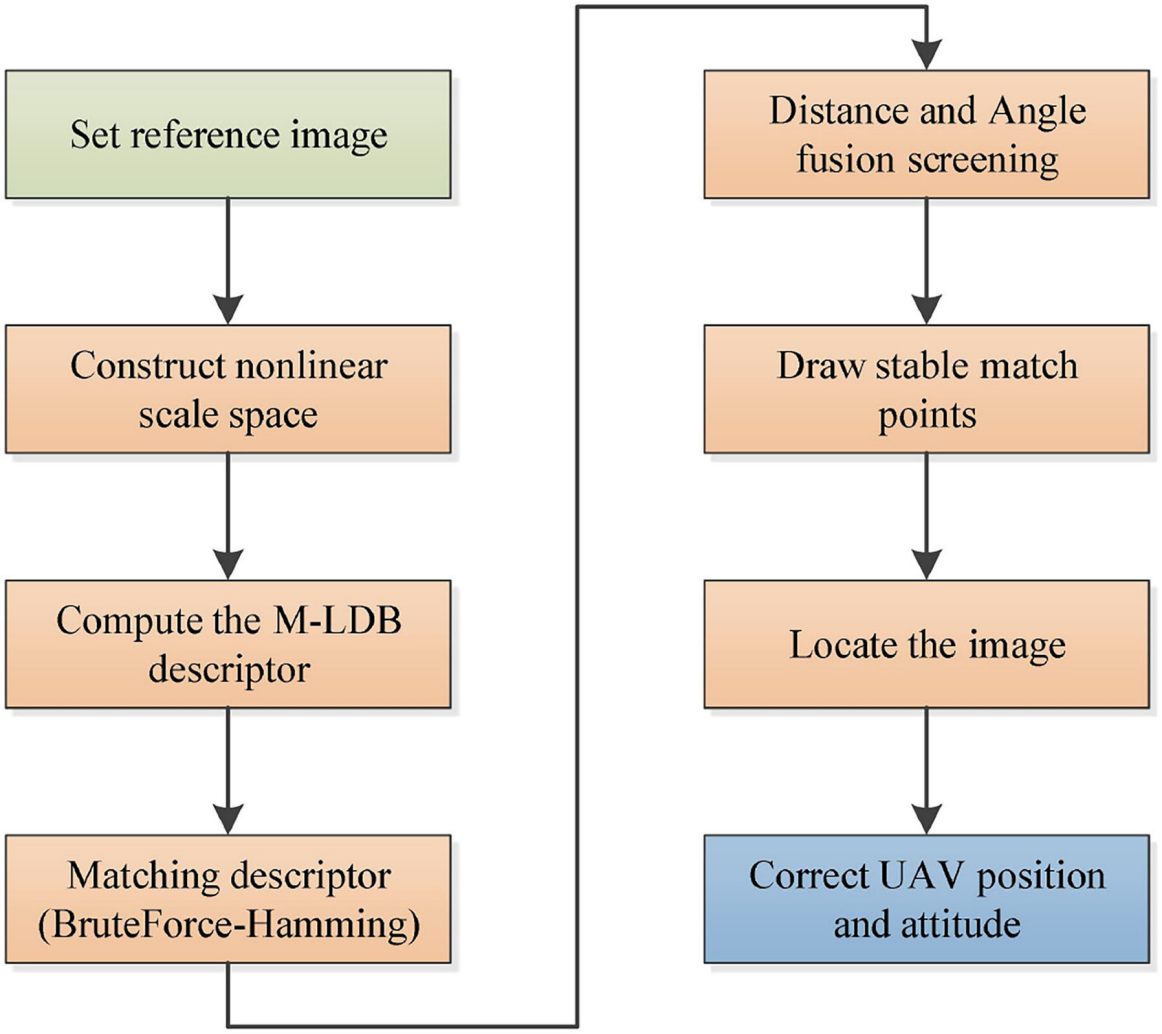
System algorithm flow chart.

Above is the algorithm flow of this experiment, and the algorithm flow chart is shown in Figure 6.

## Algorithm embedded device verification

**Figure 7:**
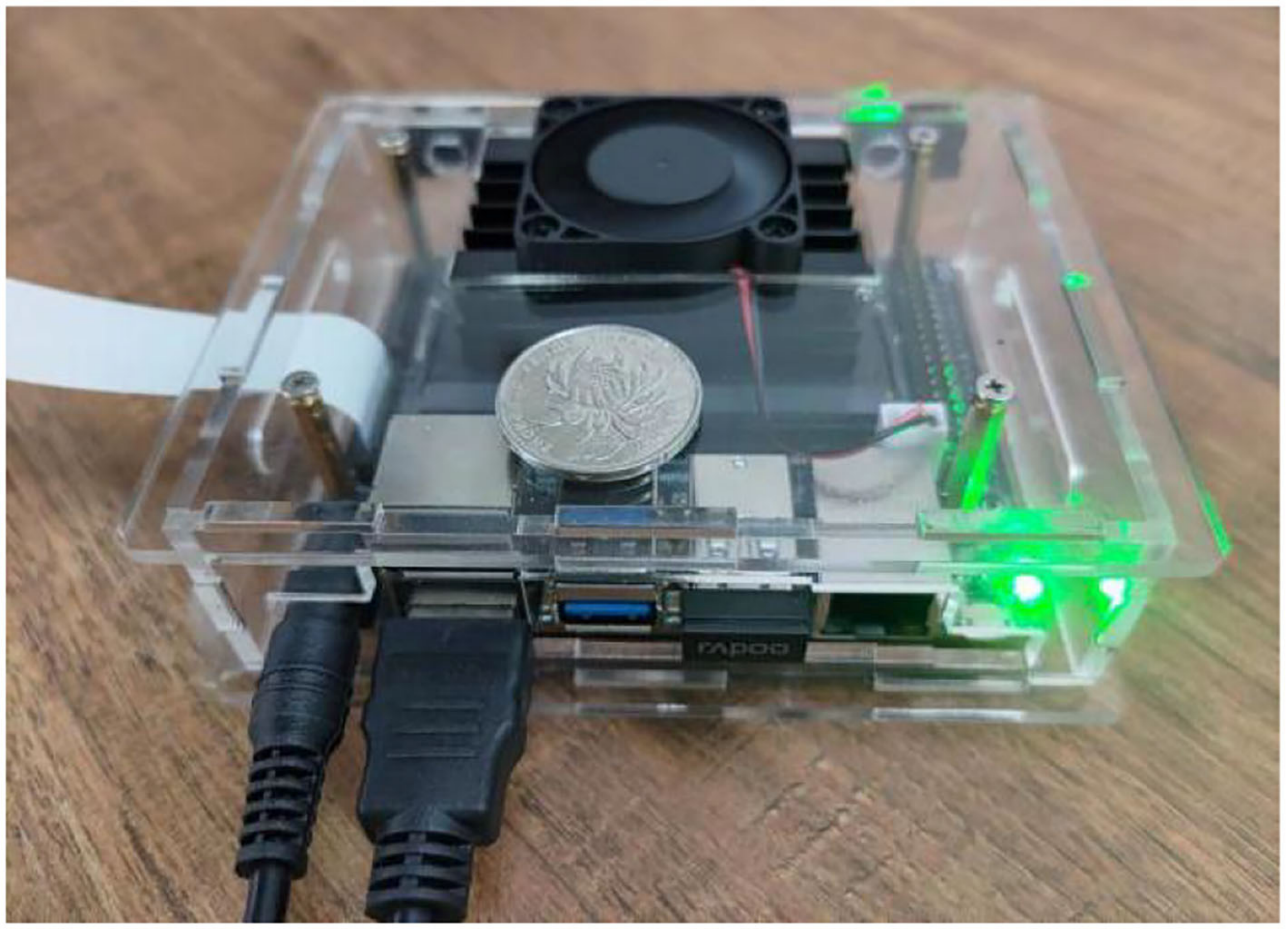
JETSON NANO B01.

As shown in Figure 7, the JETSON NANO B01 embedded computer serves as the verification platform for algorithm implementation (Kalms et al., 2017; Bao et al., 2022; Edel and Kapustin, 2022; Zhang et al., 2022). The UAV is equipped with a vision computer, and the camera is used as the image acquisition terminal to calculate the features of the real-time image and the benchmark image. The real-time image is located in the benchmark image by feature matching, and the translation, rotation, and scaling of the real-time image are calculated and input into the flight controller as compensation parameters of flight control, so as to realize the pose correction of the UAV. Based on the OpenCV 4.5.5 computer vision library, this paper applies the Qt 5.14.2 software development platform to develop visual algorithm software.

As shown in Table 1, the performance parameters of the JETSON NANO B01 are embedded into the computer for algorithm verification.

**Table 1:** The algorithm verifies the platform performance parameters.

**Item**	**Value**
Computing Power:	472 GFLOPS
CPU:	Quad-core ARM^Ⓡ^ Cortex^Ⓡ^-A57 MPCore processor
GPU:	NVIDIA Maxwell^TM^ architecture with 128 NVIDIA CUDA^Ⓡ^ cores 0.5 TFLOPS(FP16)
Memory:	4 GB 64 bits LPDDR4 1600 MHz-25.6 GB/s
Operating System:	ubuntu 18.04 LTS

In this paper, a large number of algorithms have been verified for various scenes such as ordinary road surface and grass, and the image matching localization method has been fully verified in the process of translation, rotation, and climbing of the UAV. The input image size of the visual navigation module is 640 × 480, which can realize real-time image processing (Jiang et al., 2015; Soleimani et al., 2021) and ensure the real-time demand of the flight control response cycle.

## Scenario 1: Common pavement scenario verification

(1) The UAV rotates, that is, the UAV heading changes.

**Figure 8:**
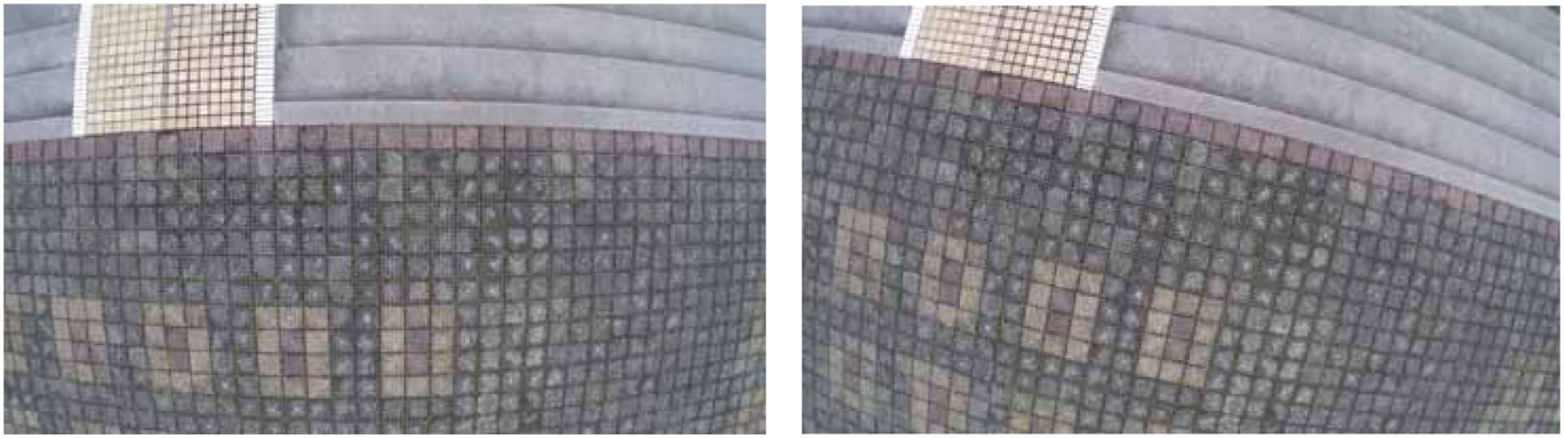
Images taken while the UAV is rotating.

**Figure 9:**
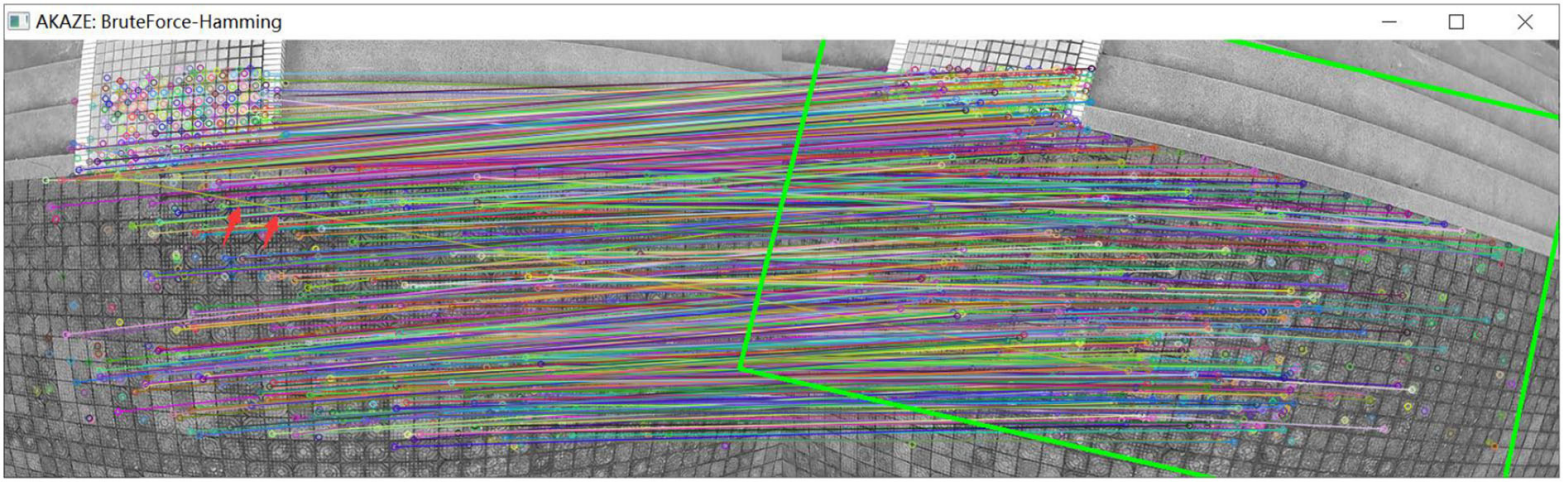
Matching effect of original algorithm.

**Figure 10:**
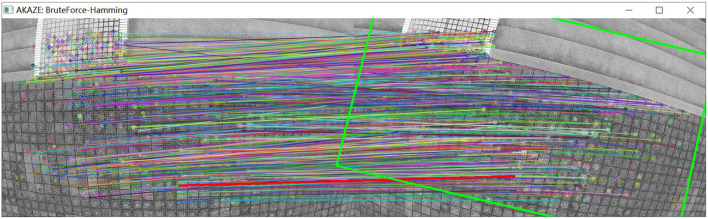
Matching effect of the optimized algorithm.

As shown in Figure 8, a set of images is captured by the camera when the UAV rotates. As shown in Figure 9, the matching and positioning results of the original AKAZE algorithm are shown. It is obvious that some wrong matching points with too large an angle deviation are seen in the figure. In this paper, an innovative method based on Hamming distance and matching line angle fusion is proposed to screen the best matching points. As shown in Figure 10, in order to obtain matching effect, the improved algorithm greatly improves the robustness of the algorithm without affecting the performance of the algorithm. The bold red line in Figure 10 is the matching line with the minimum Hamming distance, which is used as the reference line. A matching point that pairs with a too large deviation angle is eliminated through the algorithm to obtain more accurate matching results.

As shown in Table 2, are the translation (*Tx*, *Ty*), rotation (*Rot*), and scaling (*Sx*, *Sy*) of the two groups of images in Figure 8 calculated by the visual navigation module.

**Table 2:** The output calculated by the visual navigation module.

***Tx***	***Ty***	***Rot***	***Sx***	***Sy***
−1.66501	−13.6392	0.234247	0.945235	0.950384

*Tx* and *Ty* are measured in px, and others have no units of measurement.

(2) The UAV panned and climbed.

**Figure 11:**
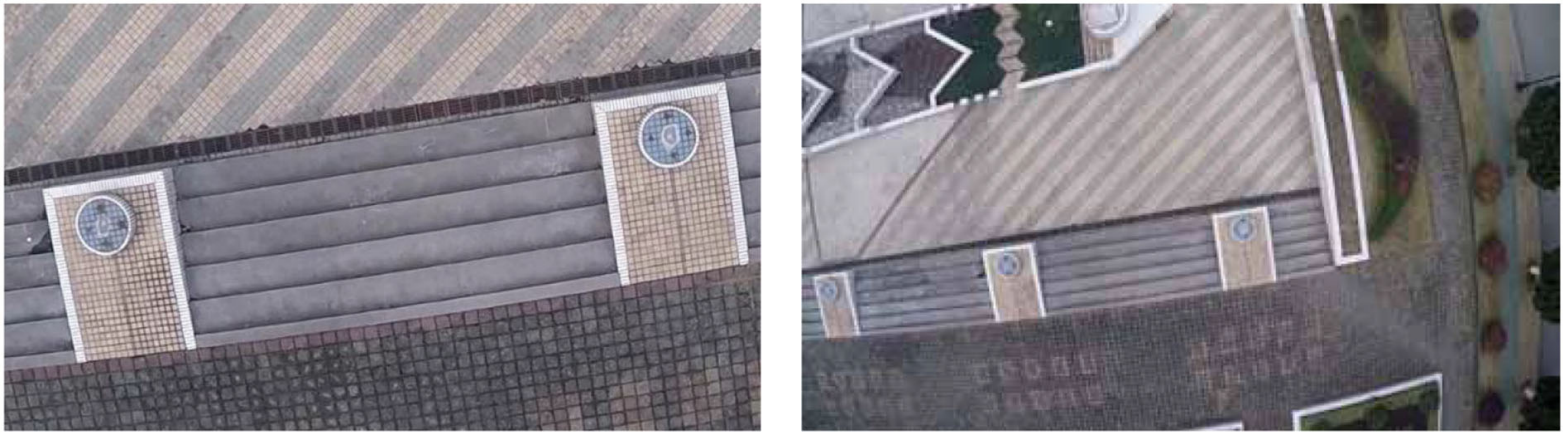
Images taken when the UAV panned and climbed.

**Figure 12:**
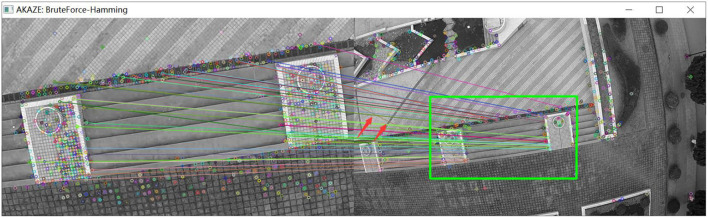
Matching effect of original algorithm.

**Figure 13:**
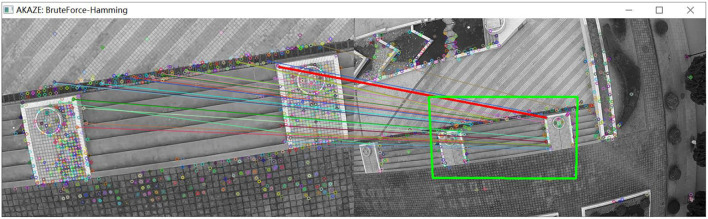
Matching effect of the optimized algorithm.

As shown in Figure 11, a group of pictures is collected by the UAV under the condition of translation and height climbing. As shown in Figure 12, through the matching point results of the original AKAZE method, the matching points with too large a deviation angle can also be obviously found, which further verifies the necessity of improving the direction of the algorithm in this paper. By fusing the angle deviation condition, the matching result as shown in Figure 13 is obtained, and the wrong matching points are eliminated effectively. The premise of UAV visual guidance is to obtain more accurate matching results. The algorithm in this paper has been repeatedly verified and has strong practicability and effectiveness, which greatly improves the robustness of the algorithm. The bold red line in Figure 13 is the matching line with the minimum Hamming distance, which is used as the base line for angle elimination condition.

As shown in Table 3, the translation (*Tx*, *Ty*), rotation (*Rot*), and scaling (*Sx*, *Sy*) of the two groups of images in Figure 11 are calculated by the visual navigation module.

**Table 3:** The output calculated by the visual navigation module.

***Tx***	***Ty***	***Rot***	***Sx***	***Sy***
−62.5681	45.6894	0.00135039	0.422023	0.409761

*Tx* and *Ty* are measured in px, and the others have no units of measurement.

The algorithm is verified by a large number of ground scenes. The above are typical processing effects of several groups of algorithms, which can achieve good image matching effects under the conditions of UAV rotation, translation, and height climbing, indicating that the improved algorithm can achieve better application effects. By calculating the deviation between the images, the navigation parameters required by the UAV can be further calculated. When used as the motion compensation parameters of the UAV, it can effectively assist the UAV to complete the visual navigation and achieve accurate fixed-point hovering.

## Scenario 2: Grassland scenario verification

(1) The UAV rotates, that is, the UAV heading changes.

**Figure 14:**
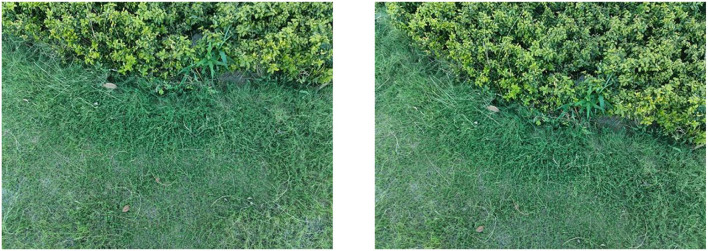
Images taken while the UAV is rotating.

**Figure 15:**
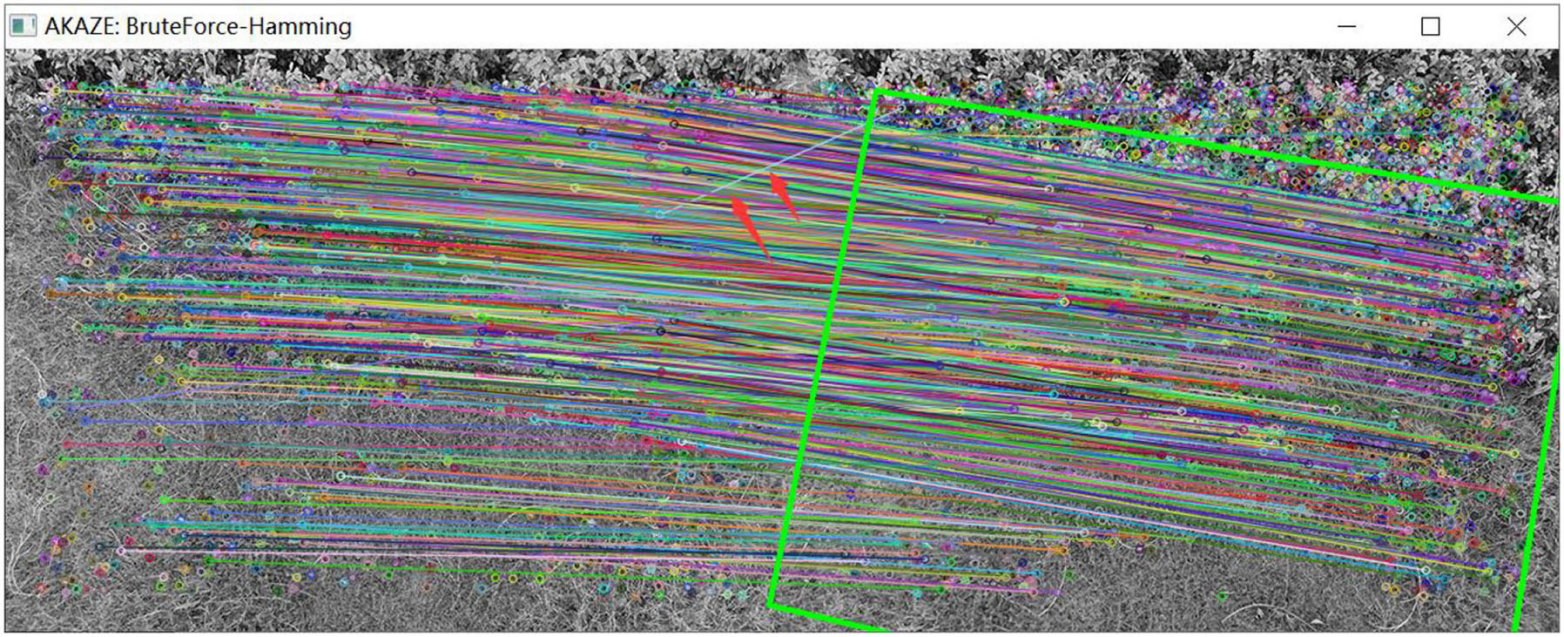
Matching effect of original algorithm.

**Figure 16:**
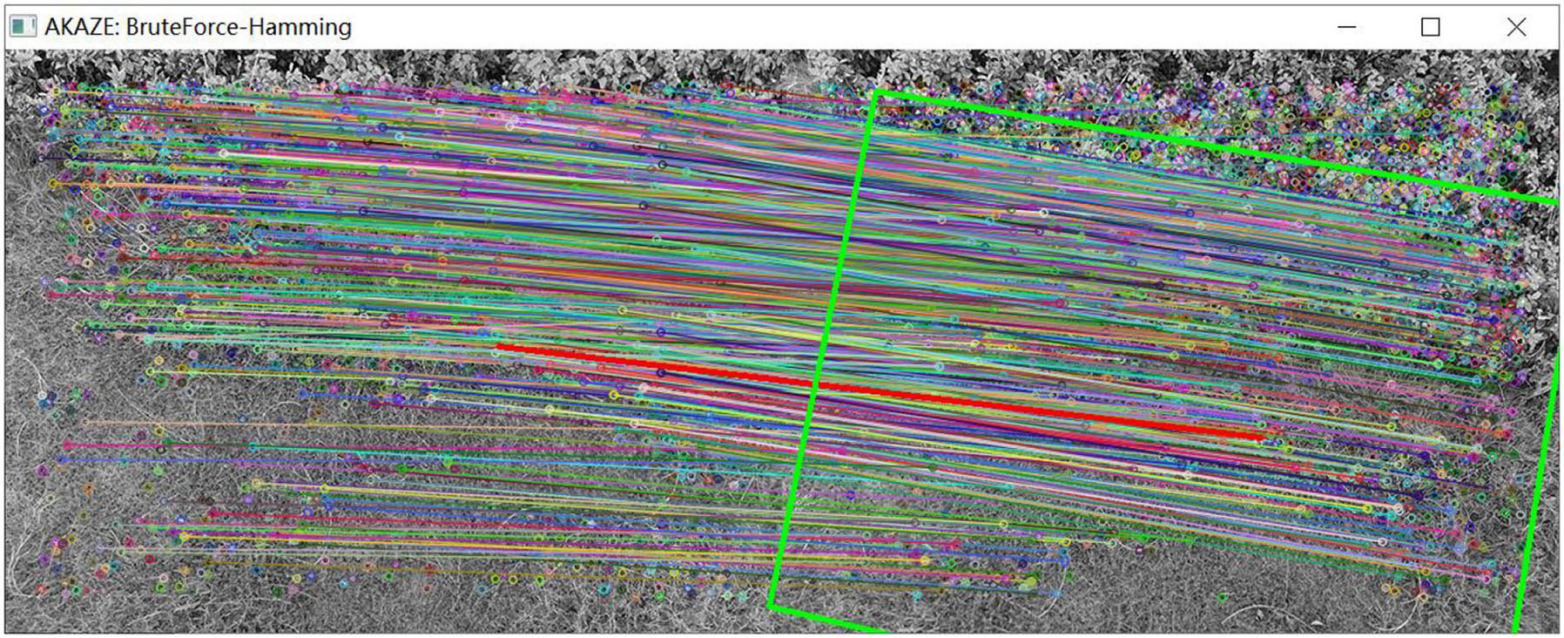
Matching effect of the optimized algorithm.

As shown in Figure 14, a set of images is captured by the camera when the UAV rotates. By detecting the features of the two images, matching of the two images is further realized. As shown in Figure 15, the matching results of the original AKAZE algorithm can obviously find some wrong matching points with too large a deviation angle, which will affect the accuracy of the subsequent calculation of motion parameters. In order to solve this problem, this paper proposes the best matching point screening method based on Hamming distance and matching line angle fusion, which achieves very good results. The matching effect of the improved algorithm is shown in Figure 16, where the bold red line is the matching line with the shortest Hamming distance, which is regarded as the base line. The matching point pairs that deviate too much from the baseline line are often wrong.

As shown in Table 4, the translation (*Tx*, *Ty*), rotation (*Rot*) and scaling, (*Sx*, *Sy*) of the two groups of images in Figure 14 are calculated by the visual navigation module.

**Table 4:** The output calculated by the visual navigation module.

***Tx***	***Ty***	***Rot***	***Sx***	***Sy***
10.4906	75.4823	0.162642	0.910785	0.883817

*Tx* and *Ty* are measured in px, and the others have no units of measurement.

(2) The UAV panned and climbed.

**Figure 17:**
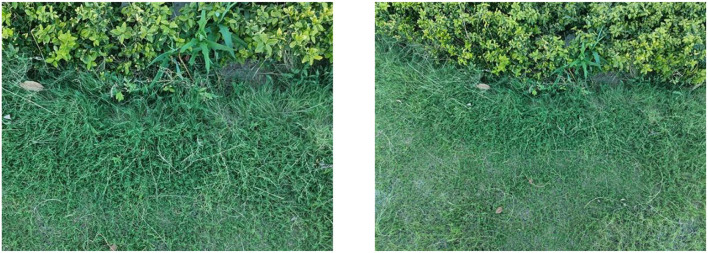
Images taken when the UAV panned and climbed.

**Figure 18:**
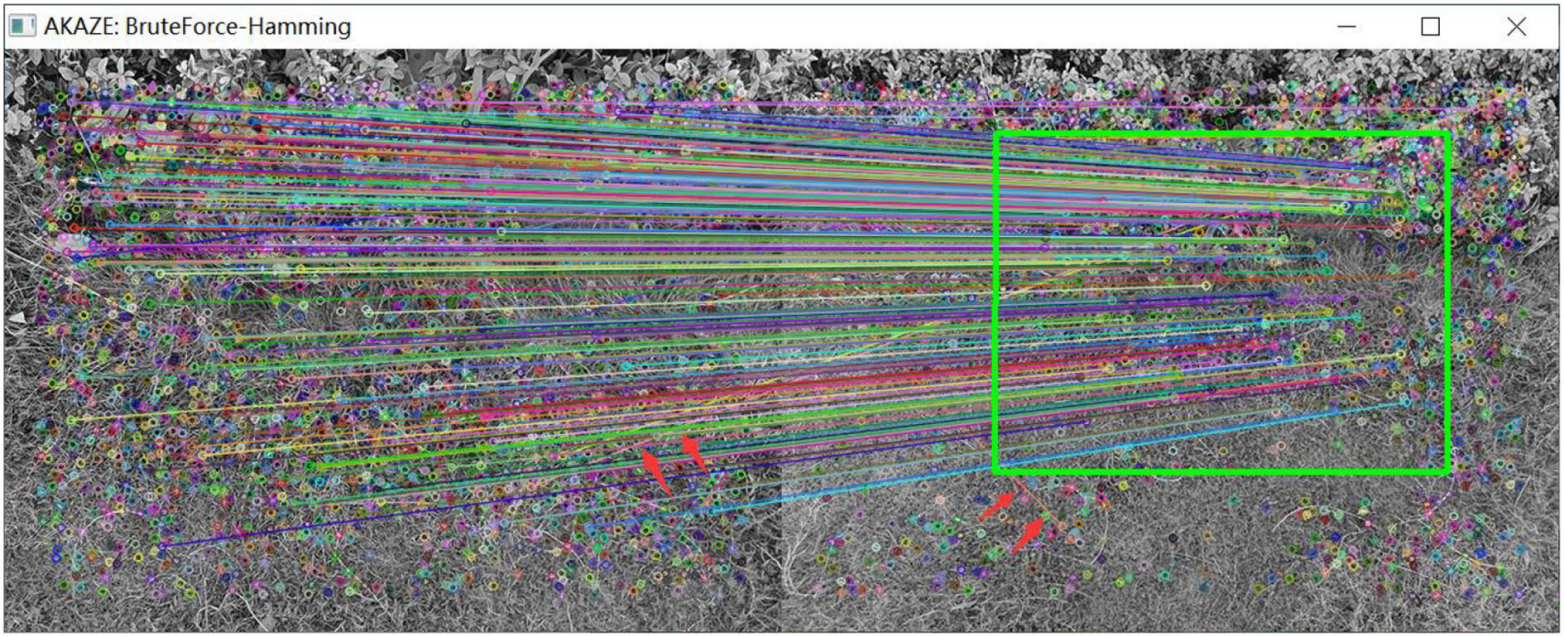
Matching effect of original algorithm.

**Figure 19:**
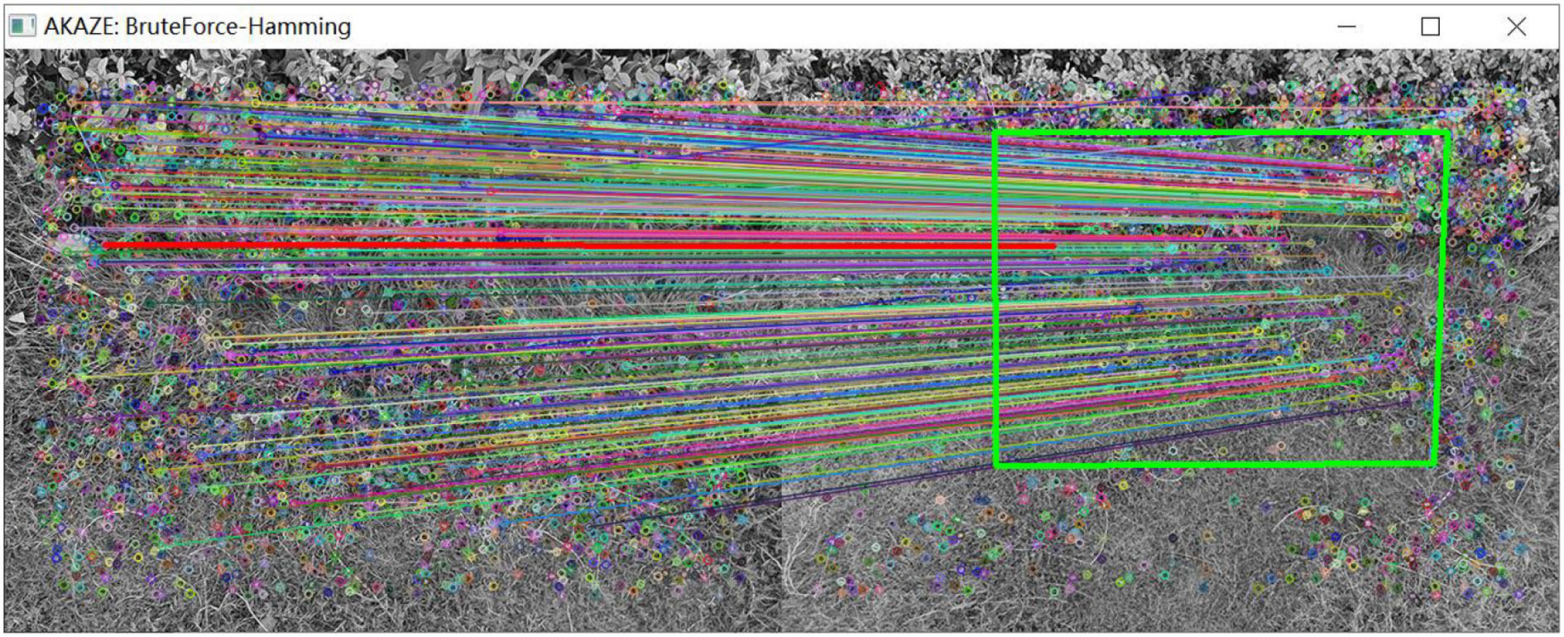
Matching effect of the optimized algorithm.

As shown in Figure 17, a group of pictures is collected by the UAV under the condition of translation and altitude climbing. As shown in Figure 18, matching results are obtained by image feature matching for the original AKAZE algorithm. The red arrow in the figure indicates that some wrong matching points with too large a deviation angle are excluded. In view of these shortcomings of the original algorithm, the matching effect as shown in Figure 19 is obtained following improvements outlined in this paper, which can effectively eliminate these obvious wrong matching points and greatly improve the robustness of the algorithm, thus laying a foundation for UAV visual guidance. In this way, more accurate matching results can be obtained, and the amount of feature deviation can be calculated more accurately, which makes the guidance of UAV hovering more accurate. In Figure 19, the bold red line is the matching line with the shortest Hamming distance, which is the benchmark reference line innovatively proposed in this paper and provides a reference for eliminating erroneous matching points.

As shown in Table 5, the translation (*Tx*, *Ty*), rotation (*Rot*), and scaling (*Sx*, *Sy*) of the two groups of images in Figure 17 are calculated by the visual navigation module.

**Table 5:** The output calculated by the visual navigation module.

***Tx***	***Ty***	***Rot***	***Sx***	***Sy***
36.2779	−35.5712	≤0.001	0.583277	0.572635

Tx and *Ty* are measured in px, and the others have no units of measurement.

Through the repeated test of the grassland scene, good experimental results are also obtained, which fully verifies that the proposed algorithm can achieve good image matching effects in multi-scenes and is suitable for multi-scene UAV visual guidance. This paper has achieved good results in the direction of improving the robustness of the AKAZE algorithm, and innovatively proposed the best matching point screening method based on Hamming distance and matching line angle fusion. A large number of experiments have been carried out on ordinary ground, grassland, and other scenes, which fully verified the feasibility and effectiveness of the algorithm.

## Conclusion

Aiming at the high-precision hovering problem of UAV operation, this paper proposes a high-precision visual positioning and orientation method of UAVs based on image feature matching. The image feature matching based on the improved AKAZE algorithm is realized, and the optimal matching point pair screening method based on the fusion of Hamming distance and matching line angle is innovatively proposed, which greatly improves the robustness of the algorithm without affecting the performance of the algorithm. Aiming at the diversity and complexity of the UAV operating environment, the application of image scale invariant features for UAV visual navigation is proposed. By setting the reference image of UAV operation, the real-time image is matched with the reference image for feature matching, and the translation, rotation angle, and scaling ratio of the image are calculated as the input parameters of the aircraft pose control, so as to reduce the image feature deviation and achieve the UAV pose state correction. A lot of tests and verification are carried out. Finally, the multi-scene UAV flight environment verification is carried out, and good application results are obtained, which shows that the algorithm has high engineering application value.

## Data availability statement

The original contributions presented in the study are included in the article/supplementary material, further inquiries can be directed to the corresponding author.

## Author contributions

BX and ZY contributed to conception and design of the study and performed the statistical analysis. BX organized the database and wrote the first draft of the manuscript. BX, LL, CZ, HX, and QZ wrote sections of the manuscript. All authors contributed to manuscript revision, read, and approved the submitted version.

## Funding

This research was funded by the Guizhou Provincial Science and Technology Projects under Grant Guizhou-Sci-Co-Supp[2020]2Y044 and in part by the Science and Technology Projects of China Southern Power Grid Co. Ltd. under Grant 066600KK52170074.

## Conflict of interest

Author BX is employed by Nanjing Taiside Intelligent Technology Co., Ltd. Author QZ is employed by Electric Power Research Institute of Guizhou Power Grid Co., Ltd. The remaining authors declare that the research was conducted in the absence of any commercial or financial relationships that could be construed as a potential conflict of interest.

## Publisher's note

All claims expressed in this article are solely those of the authors and do not necessarily represent those of their affiliated organizations, or those of the publisher, the editors and the reviewers. Any product that may be evaluated in this article, or claim that may be made by its manufacturer, is not guaranteed or endorsed by the publisher.
